# Gas Protection of Two-Dimensional Nanomaterials from High-Energy Impacts

**DOI:** 10.1038/srep35532

**Published:** 2016-10-19

**Authors:** Tan Xing, Srikanth Mateti, Lu Hua Li, Fengxian Ma, Aijun Du, Yury Gogotsi, Ying Chen

**Affiliations:** 1 Institute for Frontier Materials, Deakin University, Waurn Ponds, Victoria 3216, Australia; 2 School of Chemistry, Physics and Mechanical Engineering, Queensland University of Technology, Brisbane, QLD 4001, Australia; 3 A. J. Drexel Nanomaterials Institute, and Materials Science and Engineering Department, Drexel University 3141 Chestnut Street, Philadelphia, PA 19104, USA

## Abstract

Two-dimensional (2D) materials can be produced using ball milling with the help of liquid surfactants or solid exfoliation agents, as ball milling of bulk precursor materials usually produces nanosized particles because of high-energy impacts. Post-milling treatment is thus needed to purify the nanosheets. We show here that nanosheets of graphene, BN, and MoS_2_ can be produced by ball milling of their bulk crystals in the presence of ammonia or a hydrocarbon ethylene gas and the obtained nanosheets remain flat and maintain their single-crystalline structure with low defects density even after a long period of time; post-milling treatment is not needed. This study does not just demonstrate production of nanosheets using ball milling, but reveals surprising indestructible behaviour of 2D nanomaterials in ammonia or hydrocarbon gas under the high-energy impacts; in other milling atmospheres such as air, nitrogen or argon the same milling treatment produces nanosized particles. A systematic study reveals chemisorption of ammonia and hydrocarbon gases and chemical reactions occurring at defect sites, which heal the defects by saturating the dangling bonds. Density functional theory was used to understand the mechanism of mechanochemical reactions. Ball milling in ammonia or hydrocarbon is promising for mass-production of pure nanosheets.

Two-dimensional materials, such as graphene, boron nitride (BN), and transition metal dichalcogenides (TMD) nanosheets, have exceptional electronic, mechanical and physical properties^1^,^2^,^3^,^4^,^5^, which are appealing for both fundamental science and practical applications. With successive thickness reduction of the bulk crystals to nanometer scale, the inherent properties of these bulk materials are altered. For example, electrons in graphene behave as Dirac fermions^1^, MoS_2_ nanosheet undergoes a phase change from an indirect to direct band gap semiconductor^6^, and BN nanosheets exhibit exceptional ability to adsorb molecules^7^. These wonderful materials have been produced in large quantities using various techniques. Ball milling or grinding can be used for thickness reduction of layered materials and even for graphene and nanosheet production, but liquid surfactants or solid exfoliation agents have to be used in the milling processes as most ball milling treatments of the starting bulk crystals alone destroy material structure and introduce a great number of defects^8^,^9^,^10^. To reduce the damage, low-energy milling has been used together with various surfactant solutions (i.e. MDF, NMP)^5^,^6^,^11^,^12^. Wet milling media can effectively reduce the structural damage and prevent agglomeration events, but also bring in contaminants which are difficult to remove, consequently affecting nanosheet properties and applications. Dry milling of bulk crystals with solid exfoliation agents (such as dry CO_2_, sulphur, salt, etc.) is another successful approach. For example, dry CO_2_ was used in a ball milling process to produce graphene sheets with carboxylated edges^13^,^14^ and a significant amount of oxygen was introduced into graphene from CO_2_. Recently we reported on mechanochemical synthesis of BN nanosheets using ball milling with urea^12^. In these processes, post-milling treatment is required to remove the surfactants or the agents from nanosheets, which adds to production costs and introduces some contaminations. We report here that nanosheets of graphene, BN, and MoS_2_ can be produced by ball milling of their bulk crystals in the presence of ammonia or hydrocarbon (C_2_H_4_ or CH_4_) gas and the obtained nanosheets remain flat and maintain their single-crystalline structure with a low defect density even after a long period of milling time. For example, ball milling of graphite in C_2_H_4_ produces pure graphite nanosheets; milling of hBN in NH_3_ gas produces BN nanosheets without any contamination. Milling of MoS_2_ in NH_3_ produces the MoS_2_ nanosheets with NH_3_ adsorption and a simple low-temperature treatment can remove the adsorbed gases. More importantly, This study does not just intend to demonstrate production of nanosheets using ball milling, but reveals surprising indestructible behaviour of 2D nanomaterials in ammonia or hydrocarbon gas under the high-energy impacts; in other milling atmospheres such as air, nitrogen or argon the same milling treatment produces nanosized particles. A systematic investigation reveals that during the milling process, substantial amounts of NH_3_, C_2_H_4_ or CH_4_ gas molecules are absorbed on the nanosheets and chemical bonds are formed at the defects or edges created by high-energy milling impacts, preventing cross-linking and the fracture or folding of graphene and other 2D materials. Such special environment effect needs to be considered carefully for practical applications.

## Results and Discussion

**Figure 1:**
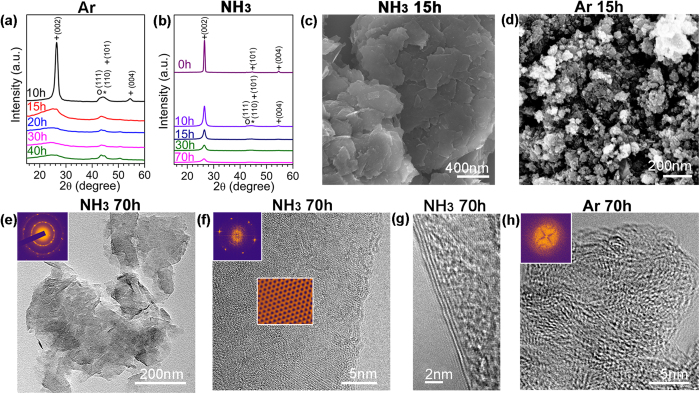
Different structural changes of graphite under ball milling in different gases. XRD patterns of graphite milled in Ar (**a**) and NH_3_ (**b**) for different times, +: graphite; O: stainless steel and *: hardened steel. SEM images showing different morphologies of graphite milled in Ar (**c**) and NH_3_ (**d**) gases. TEM images revealing different structures of the graphite milled for 70 h in NH_3_ (**e,f,g**), and in Ar (**h**). Insets show SAED patterns.

Under violent ball impacts (Figure S1), materials normally suffer from severe fracturing and plastic deformation until complete loss of the original crystalline structure^9^. For example, in the case of milling of graphite in Ar gas at 300 kPa, the X-ray diffraction (XRD) patterns in Fig. 1a show a typical gradual disordering process of the crystalline structure of graphite to full amorphization after just 20 hours of milling. Similar amorphization of graphite after ball milling has been reported by several groups previously^10^,^11^,^15^. As revealed by the XRD patterns presented in Fig. 1b, in a different milling atmosphere of ammonia (NH_3_) gas at the same pressure and milling parameters, the milling energy that destroyed the hexagonal structure of graphite in Ar gas cannot realize the same phase transformation. The graphitic structure can still be seen clearly from the XRD patterns taken from the sample after milling for 20 hours and it does not disappear even after 70 hours of extended milling. Although the intensity of the diffraction peaks decreases as milling time increases and the peaks also broaden as the result of graphite exfoliation, the XRD patterns clearly suggest that NH_3_ gas slows down or prevents disordering of graphite structure under high-energy impacts. Different structural changes in graphite in two gases were confirmed by Raman spectroscopy analysis. The Raman spectra (Figure S2) in the Supplementary Information show that the starting graphite sample has G, D, D’ and 2D bands. These bands can still be seen in the graphite after milling in NH_3_ gas for 30 hours, suggesting the same graphitic structure due to NH_3_ protection. The intensity change of G and D bands is due to the size reduction of graphite sheets^11^. In contrast, only weak and broadened G and D bands can be seen from the Raman spectra of the sample after milling in Ar for the same period of 30 hours. G and D’ bands merge and cannot be separated because of disordered structure. 2D band completely disappears. The significant difference in the Raman spectra indicates more disordered structure in the sample milled in Ar.

Different morphology changes were observed for the graphite samples milled in the two different gases. Scanning electron microscopy (SEM) images in Fig. 1 show that, in the case of milling in NH_3_, the starting micrometer-size graphite chips transform to thin layers/sheets after 15 hours of milling (Fig. 1c). The lateral sheet size is several hundred nanometers. Extended milling treatment does not change the sample morphology even after 30 hours. An entirely different morphology was found in the samples milled in Ar - round particles of less than 100 nm were produced after 15 hours of milling (Fig. 1d).

Transmission Electron Microscopy (TEM) analysis confirms different structures and morphologies of the samples milled in different gases. Figure 1e shows a typical TEM image of the graphene milled in NH_3_ for 70 hours. Most nanosheets have a thickness of a few nanometers, but few-layer graphene nanosheets are also found after centrifuging (Fig. 1g). The Selected Area Electron Diffraction (SAED) patterns contain multiple sets of dots with a six-fold symmetry (inset of Fig. 1e), revealing an undamaged in-plane structure of the nanosheets^16^. The high resolution (HR) TEM image in Fig. 1f shows a good crystallinity of a single nanosheet. The Fast Fourier Transformation (FFT) (inset of Fig. 1f) shows a set of dots in the hexagonal pattern, indicating that the individual nanosheet has a single-crystal structure. The well-retained crystalline structure can be seen from the reversed FFT image in Fig. 1f. The XRD, SEM and TEM results confirm that the high-energy ball milling in NH_3_ gas exfoliates graphite particles into thin nanosheets without destroying their in-plane structure. In stark contrast, the ball milling in Ar gas for the same time produces smaller particles of disordered (amorphous) structure, as shown by the TEM image in Fig. 1h and the corresponding diffraction pattern (inset).

**Figure 2:**
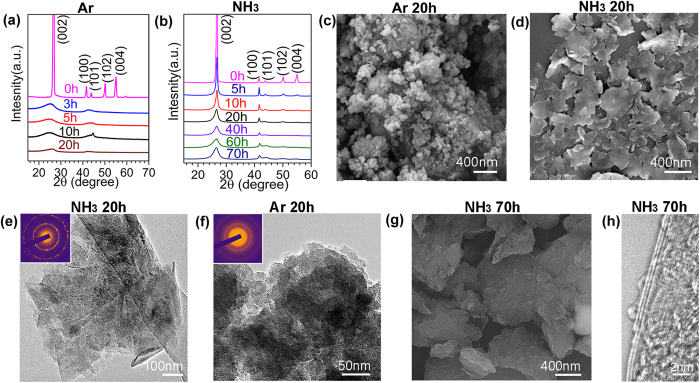
Different structural changes of boron nitride (BN) during ball milling in different gases. XRD patterns of hexagonal BN milled in Ar (**a**) and NH_3_ (**b**) for different time. SEM images of BN sample milled for 20 h in Ar (**c**) and in NH_3_ (**d**); TEM images of BN samples after milling in NH_3_ (**e**) and in Ar (**f**); SEM image of BN milled for 70 h in NH_3_ (**g**) and its TEM image (**h**).

To check if the same approach would work for other materials, hexagonal (graphitic) boron nitride (h-BN) powder was also milled under the same conditions in the two gases. The XRD patterns of h-BN in Fig. 2a,b show a similar trend as graphite. Amorphization can be seen in BN after milling in Ar for just 20 hours, while the hexagonal structure of BN can be clearly seen from the pronounced diffraction peaks even after 70 hours of milling in a NH_3_ environment (Fig. 2b). The same differences in morphologies can be seen in the corresponding SEM images in Fig. 2c,d. BN nanosheets are produced after 20 hours of milling in NH_3_ and fine nano-sized particles are the end-product of milling in Ar under the same conditions for the same period of time. TEM analyses confirm that the BN nanosheets produced by milling in NH_3_ have excellent hexagonal structure (Fig. 2e), while the milling in Ar resulted in amorphous nanoparticles (Fig. 2f), similar to the case of graphite. Extended milling up to 70 hours did not destroy the nanosheet structure in NH_3_ atmosphere as revealed by the SEM and TEM images in Fig. 2g,h, respectively.

**Figure 3:**
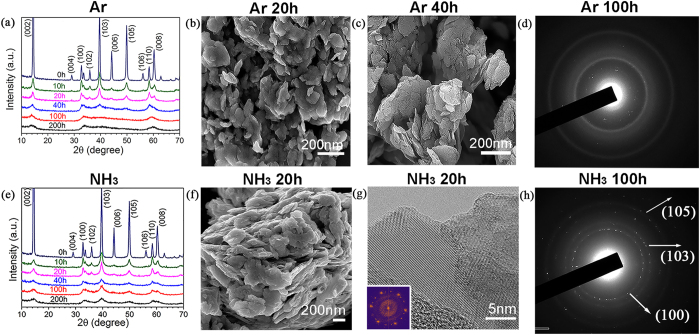
Different structural changes of molybdenum disulfide (MoS_2_) during ball milling in different gases (**a**) XRD patterns of MoS_2_ after milling in Ar gas for different periods of time; (**b,c**) SEM images of MoS_2_ after milling in Ar gas for different periods of time; (**d**) TEM micro-diffraction pattern of the sample after milling in Ar for 100 hours; (**e**) XRD patterns of MoS_2_ after milling in NH_3_ gas for different periods of time; (**f**) SEM image of MoS_2_ after milling in NH_3_ gas for 20 hours; (**g**) TEM image of MoS_2_ milled for 20 hours in NH_3_. Inset shows SAED pattern. (**h**) TEM microdiffraction pattern of the sample after milling in NH_3_ for 100 hours.

Figure 3 shows that nanoplatelets of MoS_2_ are produced in both gases, Ar and NH_3_, after milling for 20 hours and the morphology does not change after 40 hours of milling, which is consistent with the similar XRD patterns of the samples milled for 40 hours or less (Fig. 3a,e). The TEM image in Fig. 3g shows a thin layer with crystalline structure. Thus, 3 atomic layers thick MoS_2_ is more resilient to structure damage. MoS_2_ has a lesser tendency to cross-linking, as S–S bonds are less strong than Mo-S bonds. However, during further milling up to 100 hours, different structures and morphologies still can be seen. Comparing the XRD patterns of the samples milled in Ar (Fig. 3a) and NH_3_ (Fig. 3e) for 100 hours or longer, we can see that some diffraction peaks ((103) (008) and (105)) are missing from the XRD patterns of the samples milled in Ar gas and other peaks are weaker and broader than the corresponding peaks in the patterns of the samples milled in NH_3_ gas, indicating more disordered structure in the samples milled in Ar. The SAED patterns in Fig. 3d,h, confirm that NH_3_ gas has the same protective effect on the MoS_2_ nanosheets. Nanoplatelets were produced in both Ar and NH_3_ gases after short milling time and the structure was protected by ammonia gas during prolonged milling.

The protective action of NH_3_ is more pronounced on layered materials, as the effect is less evident in Si and TiO_2_, which have typical 3D structures that are deformed by dislocation gliding (Si) and brittle fracture (both) rather than shearing of layers. Their structure changes are almost the same after milling in Ar and NH_3_ gases (Supplementary Figure S3). In these cases, no nanosheets were produced. Si and TiO_2_ after milling in NH_3_ gas for 20 hours have the specific surface areas of 15.3 and 19.3 m^2^/g, respectively.

**Figure 4:**
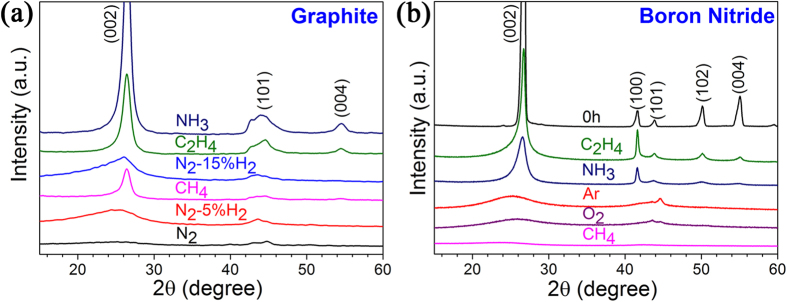
XRD patterns of graphite (**a**) and boron nitride (**b**) milled in different gases for 20 hours.

Several different gases have been tested under the same milling conditions and the XRD patterns in Fig. 4a show that the graphite samples milled for 20 hours in C_2_H_4_ and CH_4_ have sharp (0 0 2) diffraction peaks. The (0 0 4) diffraction, observable at about 55°, indicates good ordering in *c* direction. On the other hand, ball milling in N_2_ and N_2_/H_2_ mixtures does not show the same effect, leading to very wide (0 0 2) diffraction peaks, similar to the samples milled in Ar. Therefore, C_2_H_4_ and CH_4_ behave in a similar way as NH_3_, while N_2_ and N_2_ + H_2_ are like Ar and do not provide protection. SEM analysis confirms the formation of nanosheets after milling graphite in C_2_H_4_ and CH_4_ gases. For BN, milling in C_2_H_4_ gas also produces BN nanosheets, but CH_4_ and O_2_ act like Ar gas and the corresponding XRD patterns are presented in Fig. 4b. Ong and Yang observed different structure change in the milled graphite, and oxygen gas was found to protect the graphite structure^17^. Because different milling conditions were used, the different results with oxygen can be explained by much stronger interactions and, possibly, bridging of 2D layers by oxygen atoms or creation of a large number of defects due to reaction with oxygen upon high-energy milling. The XRD patterns in Fig. 4a also reveal the possible role of reactive hydrogen gas. It has been reported that pure hydrogen gas at the very high pressure of 6 MPa could have a protective effect during ball milling of graphite^18^. In the cases of milling in NH_3_, C_2_H_4_ and CH_4_ gas, full decomposition of these gases into hydrogen gas under high-energy impact (local heating) did not take place substantially because the gas pressure remains low in the sealed milling chamber during the milling. Apparently, reactions happened only at active sites where dangling bonds are created as a result of milling. Milling experiments in the mixture of N_2_ and H_2_, which were conducted to clarify the hydrogen effect, show that after only 20 hours of milling in the presence of 15% of H_2_, the (0 0 2) peak becomes wider than the one of the sample milled in NH_3_ for 70 hours (Fig. 1b). However, comparison of the XRD patterns of the samples milled in N_2_, N_2_ + 5% H_2_ and N_2_ + 15% gases, shows that the (0 0 2) broadening decreases with an increase in the H_2_ content in the atmosphere. Thus, hydrogen gas may have some protective effect but not as significant as NH_3_.

**Figure 5:**
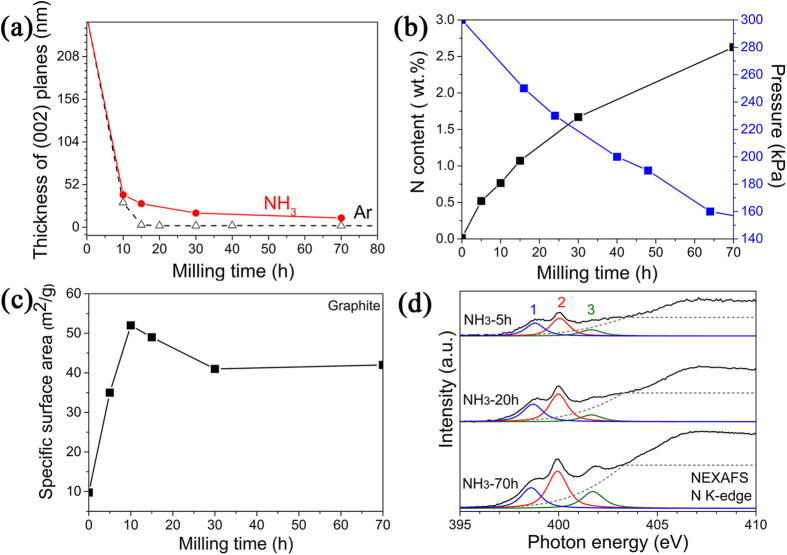
Characterization of graphite after milling. (**a**) Graphite grain-size reduction during ball milling in two different gases. (**b**) NH_3_ pressure changes during ball milling and the N content in the samples milled in NH_3_ for different times. (**c**) BET surface area change of graphite milled in NH_3_ as a function of milling time. (**d**) N K-edge NEXAFS spectra of graphite milled in NH_3_ for different periods of time.

Careful analysis of the XRD patterns finds that, for bulk (micrometer-sized) materials, different milling atmospheres apparently do not have a noticeable effect at the beginning of the milling, when the concentration of defects in the material is still low. Figure 5a shows the crystal size reduction as a function of the milling time, derived from the diffraction peak width of (002) planes in Fig. 1a,b. One can see that the grain size of graphite is reduced sharply within the first 10 hours in both gases and there is almost no difference between them until the flake thickness decreases to about 50 nm. During further milling, the nanosheets formed and became thinner gradually in NH_3_ gas; while in Ar gas, the graphite particle size continues to drop. Clearly, some gases promote the formation of nanosheets and also protect them from the damage caused by high-energy ball milling.

A significant pressure drop of NH_3_ gas from 300 kPa to 160 KPa was observed inside the sealed milling chamber during the entire milling process for 70 hours, as plotted in Fig. 5b, but no pressure change was observed in Ar gas. The pressure reduction could be explained by gas absorption onto the newly-created surfaces, which is confirmed by the nitrogen presence, with the content increasing gradually up to 2.6 wt.% in the milled samples (Fig. 5b). Ammonia pressure drop was also observed during milling of other materials (see Supplementary Figure S4). However, the continuous pressure reduction does not correlate with the surface area change over the milling process. Figure 5c shows that the surface area of graphite increases rapidly at the beginning of the milling and reaches a maximum value of about 52 m^2^/g after 10 hours and then drops down to 43 m^2^/g because of the formation of agglomerates under milling impacts^10^,^11^. The surface area results suggest that nanosheets have been produced after 10–15 hours of milling treatment, indicating an efficient production process. The surface area remains approximately constant during further milling up to 70 hours, while the NH_3_ gas pressure decreases continuously suggesting chemisorption on carbon. The chemisorption of NH_3_ molecules might happen, especially during further milling. Heating of the milled samples in the thermal gravimetric analyser (TGA) in Ar gas flow was conducted to test the absorption nature (Supplementary Figure S5). The gas molecules physisorbed on the surface can be removed under 200 °C, but the sample milled in NH_3_ was degassing up to 350 °C as indicated by the additional weight loss of 3.2 wt% above 200 °C. Extra NH_3_ might be chemisorbed on the edges or vacancies created by ball milling. The broken edges of the nanosheets (TEM images are shown in Supplementary Figure S6) presumably act as preferred sites for chemisorption of gas molecules with formation of strong chemical bonds. The TGA results as well as the continuous pressure reduction of ammonia gas over the whole milling process indicate a very high gas adsorption taking place on the nanosheets, which might play an important role for protecting the nanosheet structure and morphology. Further analysis, using near-edge X-ray absorption fine structures (NEXAFS) spectroscopy, was conducted to find possible attachment of amine or nitrogen on graphite nanosheets. Figure 5d shows the N K-edge NEXAFS spectra of the graphite after milling in NH_3_ for 5, 20 and 70 hours, respectively. Each spectrum has three relatively sharp π* resonances and broad σ* peaks at higher energies. The three π* resonances represent nitrogen atoms in four possible chemical environments. From low to high energy, they are pyridinic nitrogen at 398.7 eV (blue peak 1), pyrrolic nitrogen and amine both at 399.9 eV (red peak 2), and graphitic nitrogen at 401.4 eV (green peak 3)^19^,^20^. Although it is difficult to distinguish pyrrolic nitrogen from amine due to their similar energies, the comparison between the spectra of the graphite milled in NH_3_ for different times implies that the intensities of the three sub-peaks from graphite milled in NH_3_ increases with the milling process, which is consistent with the nitrogen testing results in Fig. 5b. The stronger peaks of pyrrolic nitrogen/amine and graphitic nitrogen suggest that NH_3_ molecules are decomposed, producing amine and nitrogen groups on carbon.

**Figure 6:**
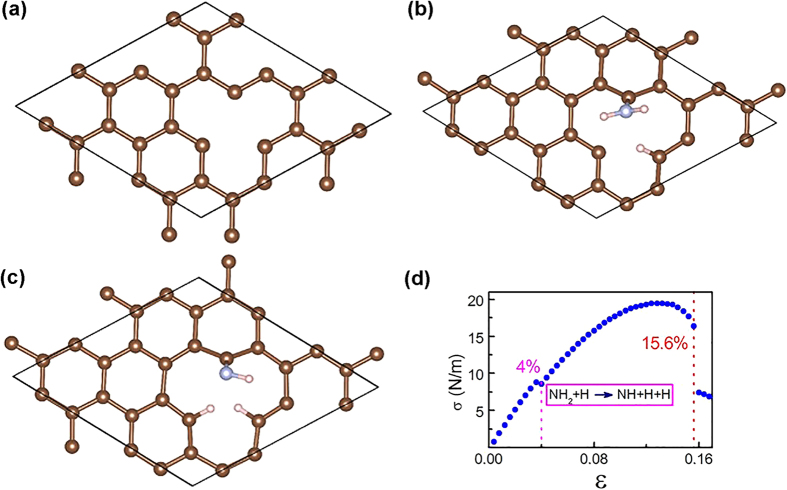
DFT modeling of amine terminations on graphene. **(a)** A defective graphene model used in the simulation. (**b**) The attached NH_2_ and H configurations decomposed from a NH_3_ molecule on the defect in graphene at 1% strain. (**c**) The attached NH, H and H configurations from the NH_3_ decomposed on the defect graphene at 4% strain. **(d)** Stress-strain curve for a defective graphene with NH_3_ attachment.

The influence of this functionalization on mechanical strength of nanosheets was investigated theoretically using Density Functional Theory (DFT) on a graphene sheet in different gases (N_2_ and NH_3_). Figure 6a presents a defective graphene model (a single atom vacancy in a graphene sheet) used to calculate the mechanical properties. Figure 6d plots the change of stress as a function of biaxial strain for the defective graphene in the presence of the adsorbed NH_3_. The calculation shows that the critical strain required for fracturing defective graphene is around 14%, but is decreased to 13.2% with N_2_ adsorption. In contrast, the critical strain required for fracturing defective graphene with NH_3_ absorption at the same site increases to 15.6%. To explore the remarkable difference of the mechanical strength of the graphene sheets with N_2_ and NH_3_ attachment, the adsorption configurations for N_2_ and NH_3_ on a defective graphene at different strains were examined. The calculation results show that the adsorption thermodynamics and kinetics for the NH_3_ attachment on a defective graphene can be significantly different under the strain. Under 1% strain, the absorbed NH_3_ molecule is first dissociated into NH_2_ and H radicals (Fig. 6b) and then the NH_2_ group is further dissociated into NH and H atoms without activation barrier when the strain is increased to 4%, confirming a mechanochemical reaction (Fig. 6c). All carbon atoms at edge sites with unsaturated bonds are saturated by the dissociated NH and H atoms. In contrast, N_2_ remains physisorbed on the defective graphene without dissociation of N_2_ molecules till fracture because of a large bonding energy in triple N≡N bond. So the experimentally observed difference in strength in different gases (in particular N_2_ and NH_3_) can be attributed to the large difference between adsorption of N_2_ and NH_3_ molecules and their interactions with carbon. Mechanochemical processes certainly play a role in these cases. Similar to the adsorption of NH_3_ on defective graphene, the attachment of NH_3_ also enhances the mechanical strength in a defective BN monolayer from 13.6% to 14.4% (Supplementary Figure S7).

**Figure 7:**
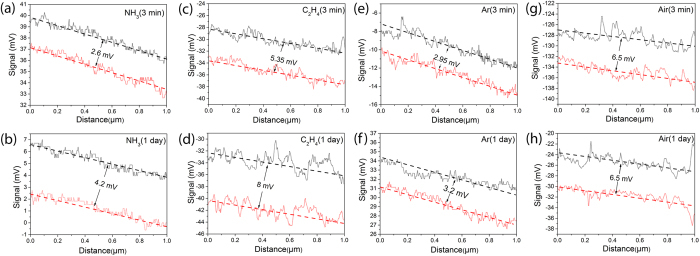
Lateral friction on BN nanosheets after removing the atmosphere of (**a,b**) NH_3_, (**c,d**) C_2_H_4_, (**e,f**) Ar, and (**g,h**) air for 3 mins and 1 day, are compared using lateral force microscopy (LFM). The black and red lines represent the torsion of the cantilever during trace and retrace in LFM scans, namely the degree of cantilever torsion due to friction recorded on photodiode in mV can qualitatively reveal the surface friction.

Possible lubrication effect of NH_3_ on BN nanosheets was also investigated by measuring the surface friction signal of nanosheets in various gases with lateral force microscopy. Because the atomic force microscope used cannot measure the friction in NH_3_ atmosphere *in situ*, a BN nanosheet was first exposed to NH_3_ gas at a pressure of 350 kPa for 24 hours and then taken out for friction signal measurement immediately under ambient condition (Fig. 7a), a second measurement was conducted after 24 hours on the same sample to see the friction change (Fig. 7b). The surface friction signal of a BN nanosheet increases from 2.6 to 4.2 mV (about 60% greater) after left in air for 1 day. The increased friction might be due to the release of NH_3_ gas from the nanosheet surface after exposing in air during 24 hours. These results indicate that NH_3_ gas functions as surface lubricant and reduces the friction between BN nanosheets and the milling bodies, reducing the shearing force applied on the nanosheets in the ball milling process. The friction signal increase of the BN nanosheet surface in C_2_H_4_ is 1.96 mV. The friction signal measured in Ar was only 0.29 mV, not much change in comparing with the friction signal in air. These results explain in part the protective effect of NH_3_ and C_2_H_4_, which might be responsible for the flat and deformation free behaviour of the nanosheets under high-energy impacts; but the saturation of dangling bonds should be the main reason for preventing structural damage under high-energy impact.

Substantial dissociation of NH_3_ molecules during ball milling has been observed previously in the case of the milling of metal powders (Zr, Ti and Mg) or B in NH_3_ for mechanochemical synthesis of metal nitrides and BN nanotubes^21^,^22^. In the current case, the pressure remains low and hydrogen atoms are absorbed on the nanosheets instead of releasing into the milling chamber. Therefore, the theoretically predicted ammonia dissociation and attachment is possible. Similar mechanisms probably act when graphite is ball-milled with melamine to produce few-layer graphene flakes^23^. We also observed a similar reaction during the milling process of BN with urea^12^. The saturation of dangling bonds is the main reason for preventing structural damage under high-energy impact. If defects are created, cross-linking of graphene (or BN) layers occurs, making shearing of the graphite flakes impossible and leading to fracture and fragmentation of the material, which creates new unsaturated bonds, and so on, until a highly disordered or even amorphous structure is formed. Cross-linking of graphene layers due to defects has been observed, e.g., after irradiation of carbon nanotubes^24^.

Because of the chemisorption and mechanochemical reactions in reactive ammonia or hydrocarbon environments, nanosheets appear to be able to self-heal their structural damage, which helps the exfoliation of bulk crystals into nanosheets and also protects their in-plane structures. SEM and TEM analyses found that most nanosheets remain flat and don’t have severe plastic deformation (folding or twist) or cross links between layers. These gas molecules are chemisorbed on defects and edges, saturating dangling bonds and preventing cross-linking of graphene or BN layers^12^ and further damage. Therefore, nanosheets remain indestructible, even under high-energy ball milling conditions. DFT results support chemical bonding in the presence of ammonia. Under stress, NH_3_ can be decomposed to form NH_x_ groups that form bonds with C or B radicals in graphene or BN, respectively. In the case of N_2_ and H_2_, only physisorption takes place, but chemical bonding with C or B is difficult because of a higher energy required to break diatomic molecules (dissociation energy of N–N bond is 945 kJ/mole)^25^. Therefore, N_2_ does not show the protective effect. Decomposition of C_2_H_4_ and CH_4_ is relatively easy because of a relatively low dissociation energy of C–H bonds (400–460 kJ/mole)^25^, and thus a similar protective effect is observed. Under the same milling conditions, both chemical adsorption/bonding and surface lubrication effects should apply to all materials regardless of their structures (i.e. hexagonal or cubic). Our results show that the protective effect is more pronounced in the layered materials than the cubic materials (Si and TiO_2_) possibly because of their larger surface areas, more absorbed gases and different deformation mechanisms.

In the current case, pure graphene nanosheets have been produced by ball milling of graphite in hydrocarbon gas. In addition, BN and MoS_2_ nanosheets were successfully produced using mechanochemistry. Thus, mechanochemical treatment of layered materials provides a new general approach for mass production of nanosheets with a fairly low density of defects using a short milling time of less than 20 hours. The nanosheets can be used as solid lubricants, additive to polymers, battery electrodes and many other applications where large amounts of multilayer 2D flakes or nanosheets are needed.

## Conclusions

Nanosheets of graphene, BN, and MoS_2_ were protected by NH_3_, C_2_H_4_ and CH_4_ gases under high-energy ball milling, while amorphous or highly disordered nanoparticles were produced in Ar, N_2_, and O_2_ under the same milling conditions. 2D nanomaterials become indestructible under high-energy impacts in certain gases due to high absorption of the ammonia and hydrocarbon gases and the mechanochemical reaction of reactive gases with dangling bonds formed during milling and chemisorption of reactive species, terminating bonds and preventing the cross-linking of layers due to the formation of bridging bonds. This milling process in the reactive gas can be used to produce large quantities of different nanosheets.

## Experimental Section

The ball milling experiments were performed in a rotating high energy ball mill^26^. In a typical experiment, 4 grams of powder were loaded in the milling jar with 4 hardened steel balls weighing 66 grams each and having a diameter of 2.5 cm. The rotating speed was 150 rpm. At the beginning of the experiment, the milling jar was vacuumed and then filled with a selected gas at 300 kPa and the gas pressure was monitored using a pressure gauge installed on the chamber lid. The structure of the samples was studied with X-ray powder diffraction (XRD) using a PANalytical X’Pert Pro diffractometer (Cu K-alpha radiation, λ = 0.15418 nm). The morphologies of the samples were studied using a scanning electron microscope (SEM, Supra 55VP) and a transmission electron microscope (TEM, JEOL 2100F). The nitrogen content was measured using a LECO TC 600 Oxygen and Nitrogen Determinator. Near edge X-ray absorption fine structure (NEXAFS) analysis was conducted at the Australian Synchrotron centre with the step of photon energy of 50 meV for carbon and nitrogen edges and 20 meV for the boron edge. Software XPSPeak was used to least-fit the NEXAFS spectra. The ionization potential was determined according to previous reports, and Voigt (convolution of Gaussian and Lorentzian) functions with the same full width at half maximum (FWHM) were used in the fitting so that both instrumental and excitation life-time broadenings were included. Cypher scanning probe microscope (SPM) was used to measure the surface friction signal of samples. An Olympus Bio-Lever cantilever (spring constant = 0.005CONTR, NanoWorld) was used to raster scan the surface of the sample in lateral force microscopy (LFM) mode, during which bending of the cantilever was recorded in mV (directly related to the friction between the cantilever and sample surface). A BN nanosheet sample was exfoliated on 90 nm silicon oxide covered silicon wafer via the scotch tape method using single crystal hBN. The silicon wafer and BN particles were placed inside the sealed chamber of selected atmosphere with 350 kPa pressure for 1 day. It was transferred to the SPM and the friction signal was measured on a flat flake of sample of approximately 10 μm ∗ 5 μm after different periods of time up to 1 day. The difference of two measurements was the friction deviation of the surface in the selected atmosphere and in air.

Calculations of the mechanical properties of the nanosheets were carried out using DFT with the projector augmented wave method^27^,^28^ and the PBE exchange correlation functional^29^ as implemented in the plane-wave basis Vienna Ab-initio Simulation Package (VASP) code^30^,^31^. A dispersion correction^32^ was incorporated to account for the long-range van der Waals interaction. An energy cut-off of 500 eV was used for the plane-wave expansion and a Monkhorst-Pack k-point mesh of 5 × 5 × 1 was used for sampling the first Brillouin zone. A supercell containing a single layer of defective graphene with 23 carbon atoms and gas molecules (N_2_ or NH_3_), and 20 Å of vacuum is used to avoid the interaction between periodical images. All the geometric structures were fully relaxed until energy and force were converged to 10^−5^ eV and 0.005 eV/Å, respectively. An in-planar biaxial strain (ε) was added along the direction of lattice vector *a* and *b* from 0 to 20%. Here ε = *a*/*a*_0_ − 1, where *a* and *a*_0_ are the strained and the equilibrium lattice constants of defective graphene, respectively.

## Additional Information

**How to cite this article**: Xing, T. *et al*. Gas Protection of Two-Dimensional Nanomaterials from High-Energy Impacts. *Sci. Rep.* **6**, 35532; doi: 10.1038/srep35532 (2016).

## Supplementary Material

Supplementary Information
