# Supplementary material for: Gas Protection of Two-Dimensional Nanomaterials from High-Energy Impacts
Source: Sci Rep. 2016 Oct 19;6:35532. doi: 10.1038/srep35532 (PMC5069545; doi:10.1038/srep35532)
Supplement: Supplementary Information [file srep35532-s1.pdf]

Supplementary Information

Gas Protection of Two-Dimensional Nanomaterials from High-Energy Impacts

Tan Xing[1#], Srikanth Mateti[1#], Luhua Li[1], Fengxian Ma[2], Aijun Du[2], Yury Gogotsi[3] and Ying Chen[1*]

[1]Institute for Frontier Materials, Deakin University, Waurn Ponds, Victoria 3216, Australia

[2] School of Chemistry, Physics and Mechanical Engineering, Queensland University of Technology, Brisbane, QLD 4001, Australia.

[3]A. J. Drexel Nanomaterials Institute, and Materials Science and Engineering Department, Drexel University 3141 Chestnut Street, Philadelphia, PA 19104 (USA)

#: these authors contribute equally; *: corresponding author

Correspondence and requests for materials should be addressed to Y.C. (email: ian.chen@deakin.edu.au.)

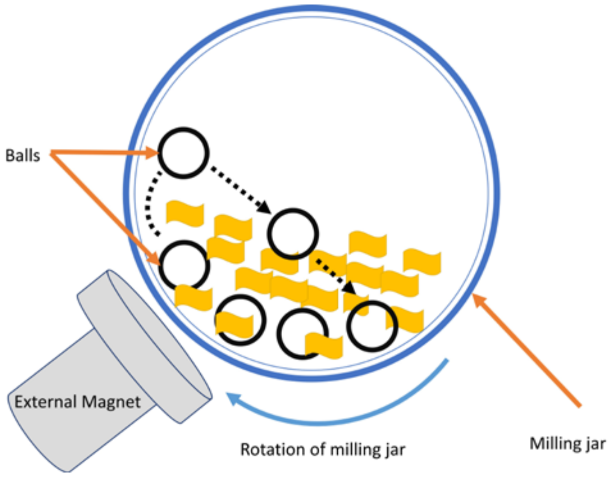

Figure S1. Schematic of high-energy ball milling device

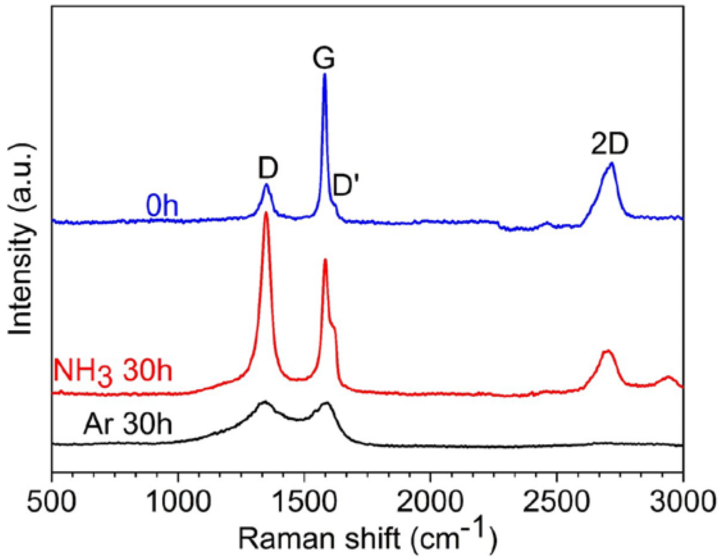

Figure S2. Raman spectra of graphite with and without ball milling treatment.

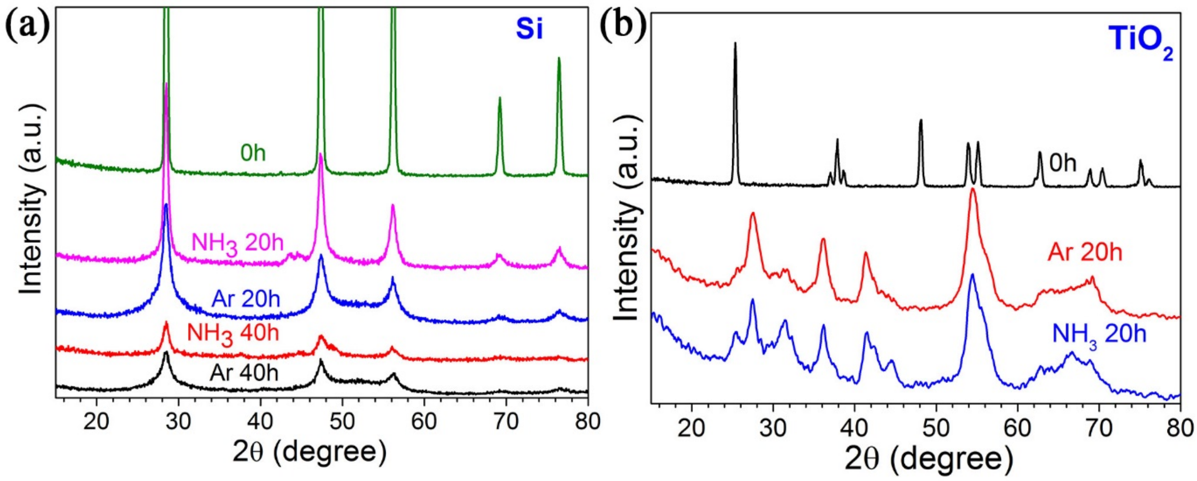

Figure S3. XRD patterns of Si (**a**) and TiO_2 (**b**) milled in different gases. The similar XRD patterns indicate the same structures produced in different gases.

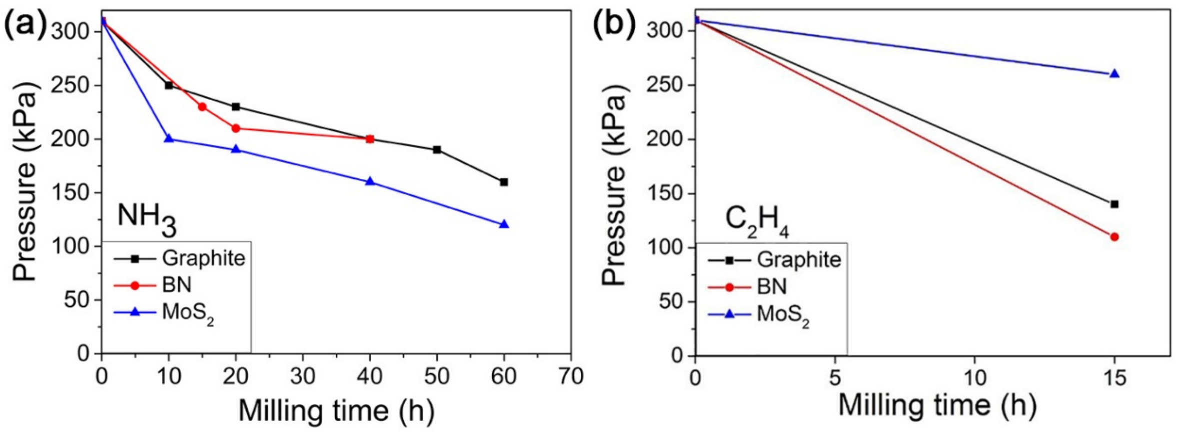

Figure S4. (**a**) NH_3 and (**b**) C_2H_4 pressure change during milling of different materials. The pressure reduction in the sealed milling chamber indicates gas absorption into the milled materials continuously over the milling process.

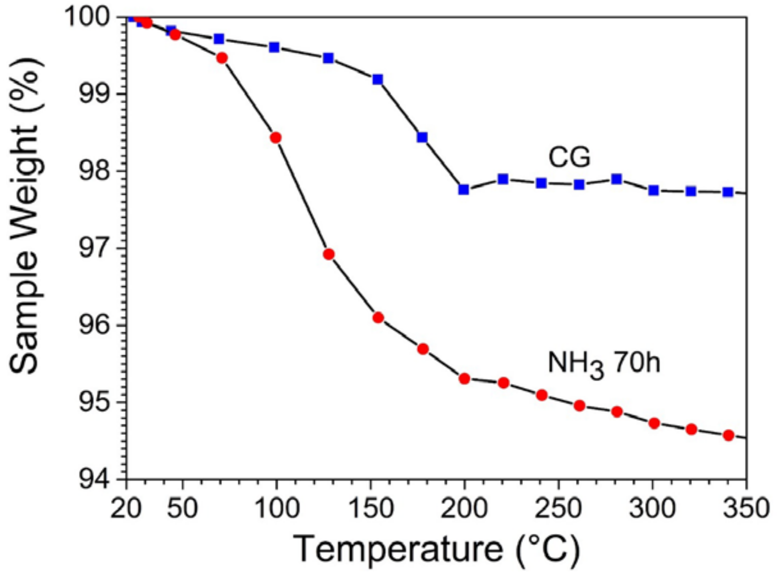

Figure S5. Thermal gravimetric (TG) curves of commercial graphite (CG) and the graphite milled for 70 h in NH3 showing release of the adsorbed gases in different temperature ranges due to their different gas adsorption (physisorption or chemisorption) nature

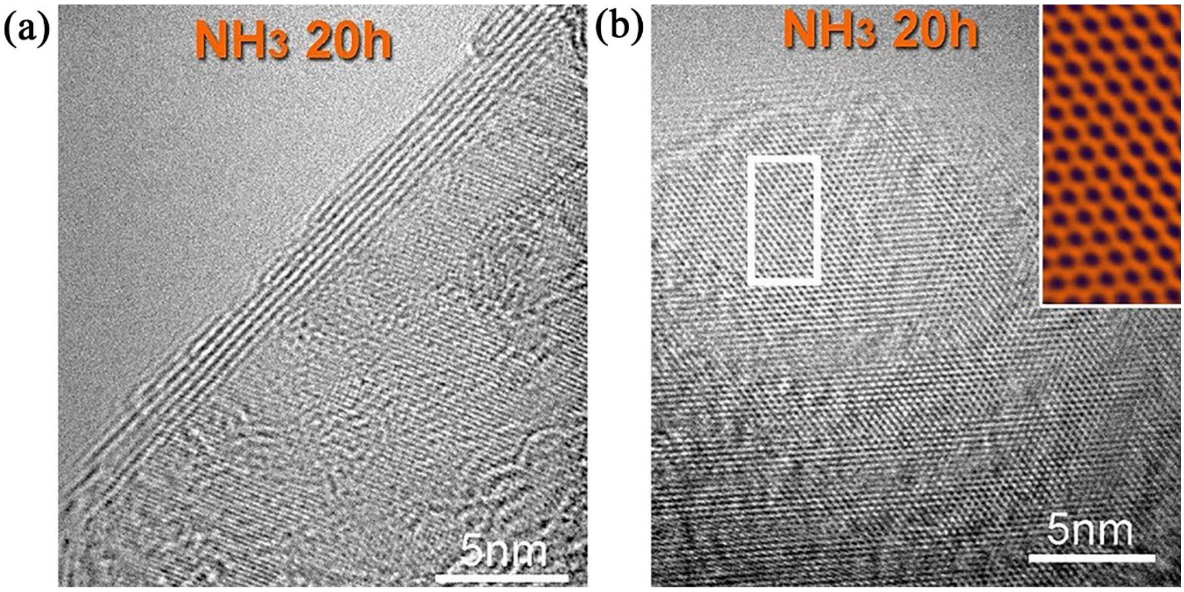

Figure S6. HR-TEM images of BN nanosheets produced by ball milling in NH_3 for 20 h. (**a**)The edges of BN nanosheets; (**b**) in-plane structure. The inset shows the hexagonal rings from the reversed FFT image of part of (**b**).

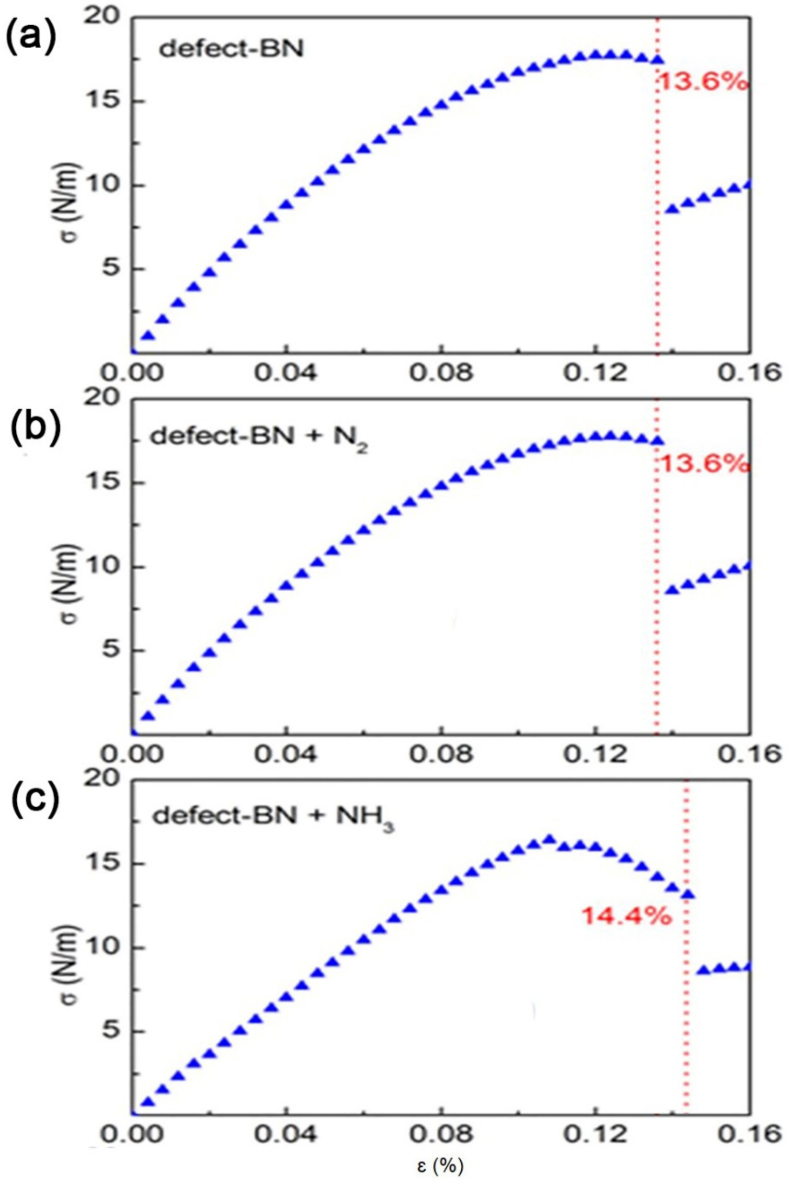

Figure S7. Simulated stress-strain curves for (**a**) a defected BN monolayer and a defected BN monolayer in the presence of (**b**) N_2 and (**c**) NH_3 attachment**.**
